# Surveillance of multidrug-resistant tuberculosis in sub-Saharan Africa through wastewater-based epidemiology

**DOI:** 10.1016/j.heliyon.2023.e18302

**Published:** 2023-07-21

**Authors:** Hlengiwe N. Mtetwa, Isaac D. Amoah, Sheena Kumari, Faizal Bux, Poovendhree Reddy

**Affiliations:** a Institute for Water and Wastewater Technology (IWWT), Durban University of Technology, PO Box 1334, Durban, 4000, South Africa; b Department of Community Health Studies, Faculty of Health Sciences, Durban University of Technology, PO Box 1334, Durban, 4000, South Africa; c Department of Environmental Science, The University of Arizona, Shantz Building Rm 4291177 E 4th St.Tucson, AZ 85721, USA

**Keywords:** Wastewater-based epidemiology, One-health approach, Multi-drug resistant tuberculosis, Molecular surveillance

## Abstract

The spread of multidrug-resistant tuberculosis (MDR-TB) is a serious public health issue, particularly in developing nations. The current methods of monitoring drug-resistant TB (DR-TB) using clinical diagnoses and hospital records are insufficient due to limited healthcare access and underreporting. This study proposes using Wastewater-Based Epidemiology (WBE) to monitor DR-TB in six African countries (Ghana, Nigeria, Kenya, Uganda, Cameroon, and South Africa) and examines the impact of treated wastewater on the spread of TB drug-resistant genes in the environment. Using droplet-digital polymerase chain reaction (ddPCR), the study evaluated untreated and treated wastewater samples in selected African countries for TB surveillance. There was a statistically significant difference in concentrations of genes conferring resistance to TB drugs in wastewater samples from the selected countries (p-value<0.05); South African samples exhibited the highest concentrations of 4.3(±2,77), 4.8(±2.96), 4.4(±3,10) and 4.7(±3,39) log copies/ml for genes conferring resistance to first-line TB drugs (*katG*, *rpoB*, *embB* and *pncA* respectively) in untreated wastewater. This may be attributed to the higher prevalence of TB/MDR-TB in SA compared to other African countries. Interestingly, genes conferring resistance to second-line TB drugs such as delamanid (*ddn* gene) and bedaquiline (*atpE* gene) were detected in relatively high concentrations (4.8(±3,67 and 3.2(±2,31 log copies/ml for ddn and *atpE* respectively) in countries, such as Cameroon, where these drugs are not part of the MDR-TB treatment regimens, perhaps due to migration or the unapproved use of these drugs in the country. The gene encoding resistance to streptomycin (*rrs* gene) was abundant in all countries, perhaps due to the common use of this antibiotic for infections other than TB. These results highlight the need for additional surveillance and monitoring, such as WBE, to gather data at a community level. Combining WBE with the One Health strategy and current TB surveillance systems can help prevent the spread of DR-TB in populations.

## Highlights

• Advanced molecular methods enable wastewater-based epidemiology in tuberculosis surveillance.
• Genes conferring resistance to tuberculosis drugs were detected in wastewater from sub-Saharan Africa.
• Genes conferring antibiotic resistance in wastewater cwere onfirmed by droplet digital polymerase chain reaction.
• South Africa and Ghana's wastewater had the highest antibiotic resistance gene levels.
• Antibiotic resistance genes in wastewater may propagate risk of tuberculosis resistance.

## Graphical abstract

**Figure:**
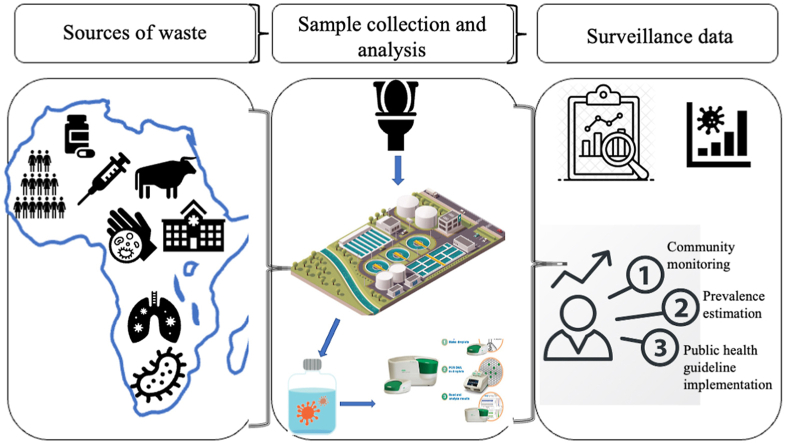

## 1 Introduction

Globally, an estimated 10.6 million people contract tuberculosis (TB) each year, resulting in 1.6 million deaths, making TB one of the top ten causes of death worldwide [1]. The global resurgence of TB, exacerbated by multi-drug resistance (MDR) of the causative agents, poses a significant challenge to disease management [2]. The dramatic increase in the TB burden in sub-Saharan Africa is a result of resource limitations especially due to the coronavirus (COVID-19) pandemic, lack of qualified of personnel, erratic drug supplies and coinfection with Human Immunodeficiency Virus (HIV) stigmatization. This stigma is associated with HIV/TB coinfection and has caused an increase in treatment defaulting, thus increasing the prevalence of drug-resistant TB. South Africa is one of the five countries with the highest burden of drug-resistant TB, with an estimated MDR-TB incidence of 23 per 100 000 people in 2019. MDR-TB is characterized by TB-causing bacteria that are resistant to at least isoniazid and rifampicin, the two most commonly used TB medications [3]. The continent also has the highest average rate of TB/HIV co-infection (31%), with co-infection rates exceeding 50% in some areas [4]. Nigeria, along with Angola, the Democratic Republic of the Congo, Ethiopia, Cameroon, Kenya, Uganda, and South Africa, constituted Africa's high TB-HIV burden countries in 2020 [[5], [6]]. In 2017, Africa alone reported 26 845 multidrug-resistant/rifampicin-resistant TB (MDR/RR-TB) cases and 867 extreme drug-resistant TB (XDR-TB) cases [4]. Before COVID-19, TB was the main priority of sub-Saharan African health authorities. In the region, 1.4 million people had TB diagnoses in 2019, but epidemiologists predicted that an additional million people were infected but went undiagnosed or untreated [5]. Therefore, a growing focus has been placed on antibiotic resistance and its environmental determinants to address the increasing healthcare crisis. The One Health framework recognizes antimicrobial resistance as a multi-domain issue with core and interconnected components in human health, animal health, and the environment [5,7]. This framework supports the need for improved surveillance methods in all of these domains and includes environmental reservoirs and resistance sources such as wastewater treatment plants in urban areas, biosolids or soil receiving manure, the atmosphere, which includes longer-distance dispersal and aquaculture production systems. Many countries' TB control strategies rely on the timely detection of cases to treat the disease effectively. Importantly, to guide therapy and prevent the spread of resistant organisms in communities, drug-resistant TB requires an accurate diagnosis of the type of resistance. Traditional culture-based TB diagnoses, on the other hand, are labour-intensive, expensive, and time-consuming and they are frequently unavailable in developing countries [5,8,9]. This makes tracking the occurrence and spread of these infections difficult. Clinical case reports, hospital admissions, and clinical surveys, which are used mostly in data collection, have proven to be difficult in developing countries as surveillance methods. The stigma of TB and HIV coinfection is a significant barrier to the management of TB and HIV, and it is one of the main social factors causing hospital delays and hindering compliance among TB patients. It is partially rooted in judgment, blame, and shame; worries about TB transmission; and public health practice and policy [10,11]. Wastewater-based epidemiology (WBE) is an approach based on the human health biomarkers excreted via faeces and urine that end up in sewage [12]. This approach has been adopted extensively for COVID-19 monitoring [[13], [14]], monitoring of diseases such as polio [15] and recently antimicrobials and resistant genes [16]. Given the prevalence of antibiotic resistance, as well as the emergence of multi-drug resistant bacterial strains and reports of their occurrence in the environment, the use of wastewater molecular surveillance as a public health strategy to combat infectious diseases is critical for monitoring TB and MDR-TB infections in communities. The proof of concept for the application of WBE for monitoring TB and MDR-TB infections was established in our earlier studies [[17], [18]]. The aim of this paper is therefore to investigate the feasibility and effectiveness of wastewater-based epidemiology (WBE) as a surveillance tool for monitoring drug resistant TB infections in selected African countries, in order to combat the global challenge of antibiotic resistance and improve disease management especially in resource-constrained regions.

## 2 Materials and methods

The following methods were adopted from the previously published study by Mtetwa et al. (2021), and modifications were implemented for this approach. The same methods were employed in their study.

### 2.1 Study location, wastewater sample collection and processing

Wastewater samples were collected from eight wastewater treatment plants (WWTPs) in six African countries (one WWTP per selected country and three WWTPs in South Africa). These countries encompass West, Central, East and Southern Africa making up a good representation of Sub-Saharan Africa. In South Africa, samples from three WWTPs in the city of Durban, in KwaZulu-Natal (KZN) province were selected. This is because KZN has one of the highest TB prevalence rates in South Africa (537 per 100 000 population a year). In this region, HIV prevalence is also the highest (27.6% among the 15–49 age group) and thus the rate of coinfections is higher. The data of the samples from the three WWTPs in KZN, were then pooled into one sample data representing South Africa. The WWTPs were chosen based on plants servicing at least 5000 people and those receiving hospital waste, as indicated below (Table 1). This method was adopted from the previous studies by Mtetwa et al. (2021); Mtetwa et al. (2022). Wastewater samples were taken on one occasion as detailed below, however, the analysis was done in triplicate.

**Table 1:** Information about the wastewater treatment facilities used in this study.

WWTP	City	WWTP's Capacity (Mℓ/d)	Remarks
South African- WWTP A	Durban	18.8	Treats wastewater from Hospital A, which has 17 affiliated clinics and provides district and regional-level health services to the population.
WWTP B	Durban	4.90	Treats wastewater from Hospital B, a medical facility that also acts as a referral hub for other clinics and hospitals.
WWTP C	Durban	70.0	Treats wastewater from the Hospital C complex, which provides specialized services for complicated TB and multidrug-resistant (MDR) TB.
Ghana	Kumasi	0.22	Treats domestic wastewater from hospitals and major animal farms including small scale poultry farms and waste from abattoir
Nigeria	Ibadan	0.817	Treats wastewater from a teaching hospital
Kenya	Kisumu	7.95	Treats wastewater from two hospitals, no input from pharmaceutical industries and no input from major animal farms
Uganda	Kampala	45	Treats wastewater from hospitals. No input from pharmaceutical industries, although, there are a number of businesses dealing in pharmaceutical products that are connected to the sewer lines. No input from major animal farms
Cameroon	Yaoundé	0.801	Treats domestic sewage from social housing and none from hospital, livestock farm or pharmaceutical industry

The Information from South Africa was sourced from Mtetwa et al. (2021); Cross and Buckley (2016); Mtetwa et al. (2022).

*Mℓ/d means megaliters per day.

In this study, 1 L of untreated (raw) and 1 L of final treated wastewater was collected from each WWTP. Thus, two 1-L samples (untreated wastewater and treated wastewater) were collected per WWTP, with many subsamples (100 mL) collected every 30 min until a total volume of 1 L was obtained. The samples were shipped from the five African countries to the laboratory in a cooler box with gel ice packs, maintained at 4 °C, and analyzed within 48 h. Samples were homogenized before analysis, and 50 mL subsamples were obtained and centrifuged at 3000 rpm for 20 min, after which the supernatant was removed and the pellets were stored for further analysis.

### 2.2 DNA extraction

Total genomic DNA was extracted from the pellet using the DNeasy Powersoil DNA extraction kit (QIAGEN), as recommended by the manufacturer. The quantity and quality of the extracted DNA were determined using an IMPLEN NanoPhotometer NP80 - All-in-One Spectrophotometer. All analyses were performed in duplicate.

### 2.3 Selection of genes conferring resistance to TB/MDR-TB antibiotics

The genes responsible for resistance to a variety of drugs used in drug-resistant TB (TB/DR-TB) treatment regimens were chosen (Mtetwa et al., 2021). The selection was based on information availability and usage in the countries chosen. Antibiotics used to treat TB infections are either classified as first- or second-line therapy regimens. Rifampicin, isoniazid, pyrazinamide and ethambutol are amongst the medications used in the first-line therapy regimen. Aminoglycosides (amikacin, kanamycin), cycloserine, bedaquiline, ethionamide, and delamanid are the drugs used under the second-line treatment regimen. Other antibiotics, such as such as streptomycin and cycloserine, are often added as part of TB treatment regimen. However, these are not exclusively used for TB treatment. In the study genes conferring resistance to these antibiotics were also included.

### 2.4 Detection and quantification of TB-drug resistance genes in wastewater

The ARGs responsible for resistance to the selected antibiotics were detected using the methods published by Mtetwa et al. (2021). The concentration of the selected ARGs in the wastewater samples was determined using droplet digital polymerase chain reaction (ddPCR). The ddPCR reaction volume of 22 μL contained 10 μL of the 2X QX200 ddPCR EvaGreen Supermix (Bio-Rad), 1 μL (20 ng/L) of template DNA, 1.25 μL of each of the forward primers (FP) and reverse primers (RP), each with a final concentration of 250 nM, and RNase/DNase free water. Droplets were created with a Bio-Rad automated droplet generator and amplified with a C1000 TouchTM Thermal Cycler (Bio-Rad). The following thermal cycling parameters were used: a first denaturation step at 95 °C for 10 min, followed by 45 cycles of denaturation at 96 °C for 45 s. The annealing temperatures varied for each primer (genes), including eis (50 °C for 60 s), *gryB*, *rrs*, *ethR* (52 °C for 60 s), *pncA*, *gyrA*, *katG*, *aptE* (54 °C for 60s), *rpoB, embB, fbiA* (60 °C for 60 s), *alr, ddn* (62 °C for 60 s) and *fgd1* (64 °C for 60 s). Prior to reading the plate, the incubation was carried out at 98 °C for 10 min (ramp rate: 2.2 °C/s) and kept at 4 °C for 30 min. After thermocycling, the ddPCR plates were read with the QX200 droplet reader (Bio-Rad). The QuantaSoft™ analysis Pro software extracted and analyzed droplet counts and amplitudes (Bio-Rad).

### 2.5 Statistical analysis

Microsoft Excel was used to obtain the descriptive statistics, and the Akaike Information Criterion (AIC) score was calculated using @Risk (Palisade Inc. USA) to perform a normality test. The Kruskal-Wallis and Dunn's Multiple Comparison tests were used to compare the concentrations of the different genes coding for TB drug resistance between the selected countries, as well as the ARGs present in each country based on normality tests. It must be noted that the data was not normally distributed, so median values were used to represent the concentrations in the various samples. The 95% confidence interval was used for all statistical tests, and a p-value of 0.05 was therefore considered statistically significant.

## 3 Results

### 3.1 Concentration of TB-related ARGs in untreated wastewater

#### 3.1.1 The concentration of genes conferring resistance to first-line TB drugs

**Fig. 1:**
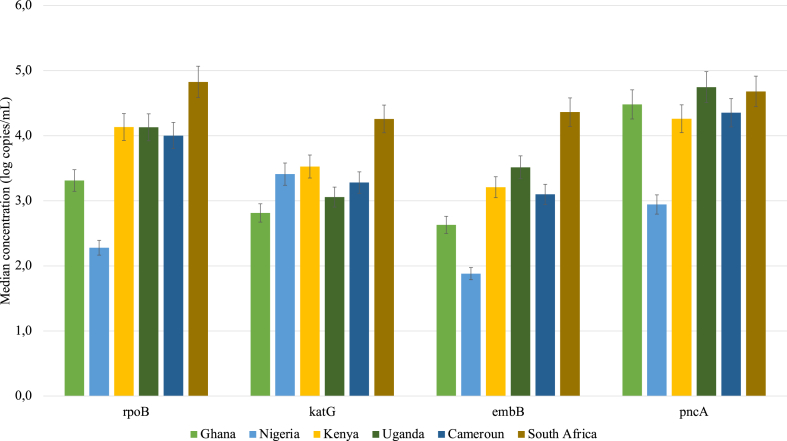
Median concentration genes encoding resistance to first-line TB drugs i.e isoniazid (*katG*), ethambutol (*embB*) rifampicin (*rpoB*), and pyrazinamide (*pncA*) in untreated wastewater from the six sub-Saharan African countries.

The highest median concentration of the genes that confer resistance to first-line TB drugs, rifampicin, isoniazid, ethambutol and pyrazinamide (*rpoB*, *katG*, *embB*, *pncA* gene), in the untreated wastewater samples (Fig. 1) were observed in samples from South Africa (4.8(±2.96) log copies/mL), (4.4(±3,10) log copies/mL, 4.7(±3,39) log copies/mL and 4.3(±2,77) log copies/mL respectively). The lowest median concentrations of *katG*, *embB,* 2.8(±2,33) log copies/mL and 1.9(±1,45) log copies/mL respectively were detected in Ghana. Furthermore, samples from Nigeria recorded the lowest median concentration of *pncA,* (2.9(±2,02) log copies/mL). In-country variations in ARG concentrations was also observed. For instance, the most abundant genes conferring resistance to first-line TB drugs in Ghana, Kenya, Uganda and Cameroun was *pncA.* However, in Nigeria and South Africa *katG* and *rpoB* genes were the most abundant respectively.

The difference in these concentrations of the first-line TB- related ARGs across all the selected countries was statistically significant for *rpoB* (p = 0.0064), *embB* (p = 0.0053), *katG* (p = 0.0234) and *pncA* (p = 0.0053). The significant variation in concentrations of *rpoB*, *katG*, *embB*, and *pncA* genes was observed between Nigeria and Uganda, Ghana and South Africa, Ghana and Nigeria, and Nigeria and South Africa respectively.

#### 3.1.2 Concentration of genes conferring resistance to second-line and other related TB drugs

**Fig. 2:**
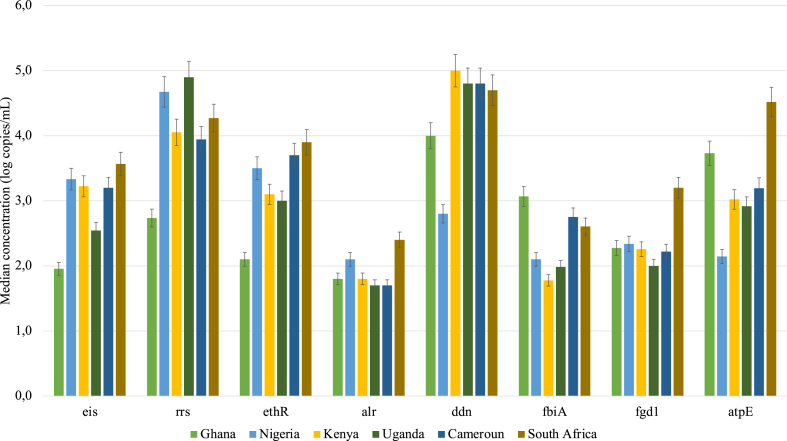
Median concentration (log copies/mL) of genes encoding resistance to second-line TB drugs i.e s streptomycin(*rrs*), aminoglycosides (*eis*), ethionamide (*ethR*), cycloserine (*alr*), delaminid (*ddn*, *fgd1* and *fbiA*) and bedaquiline (*atpE*) in untreated wastewater from six sub-Saharan African countries.

The ARGs associated with second-line TB drugs were also detected in varying concentrations in the untreated wastewater samples across all the selected countries (Fig. 2). South African samples recorded the highest concentration of ARGs associated with second-line TB treatment drugs. For instance, ARGs associated with aminoglycosides (such as kanamycin and amikacin), cycloserine, ethionamide, delamanid and bedaquiline (*eis, alr, ethR, fgd1* and *atpE* gene) had median concentrations of 3.6(±3,28) log copies/mL, 2.4(±2,02) log copies/mL, 3.9(±2,93) log copies/mL, 3.2(±1.85) log copies/mL and 4.5(±3,74) log copies/mL respectively. In contrast, the other ARGs associated with delamanid (*fbiA* and *ddn* genes) were high in samples from Ghana and Kenya respectively, with median concentrations of 3.1(±1,53) log copies/mL and 4.0(±3,44) log copies/mL respectively. The highest concentration of the ARG associated with resistance to streptomycin (*rrs* gene) of 4.9(±3,81) log copies/mL was detected in Uganda. However, the lowest concentrations of almost all the ARGs associated with second-line TB drugs (*eis, alr,* and *ethR* genes) were detected in Ghana except for delamanid (Fig. 2). *ddn* was most abundantly detected in Ghana, Uganda, Cameroon, and South Africa. These genes confer resistance to delamanid and aminoglycosides such as streptomycin respectively.

Statistically, the variation in concentrations of these second-line associated ARGs (*rrs*, *eis*, *ethR*, *ddn*, *aptE*, *fgd1*, *fbiA* and *alr*) was significant, when compared between Ghana and Uganda (p = 0.0198), Ghana and South Africa (p = 0.0115), Ghana and South Africa (p = 0.0087), Nigeria and Kenya (p = 0.0087), Nigeria and South Africa (p = 0.0087), Uganda and South Africa (p = 0.0086), Ghana and Kenya (p = 0.0197) respectively. However, no statistically significant difference (p ≥ 0.05) was found for the *alr* gene when countries were compared.

### 3.2 Concentration of TB-related ARGs in treated wastewater

The concentrations of the ARGs in treated wastewater from the selected African countries varied, showing varied contributions to environmental contamination with these ARGs. The highest median concentration for the genes encoding resistance to the first-line TB drugs observed in the treated wastewater from South Africa of 4.59(±3.86) log copies/mL for the *rpoB* gene and 4.5(±4,10) log copies/mL for the *pncA* gene, associated with the resistance to rifampicin and pyrazinamide respectively. However, the highest concentrations observed for the *katG* gene (3.7(±2,47) log copies/mL), associated with resistance to isoniazid, were detected in the treated wastewater from Nigeria and the *embB* gene, conferring resistance to ethambutol, was detected in Uganda.

**Fig. 3:**
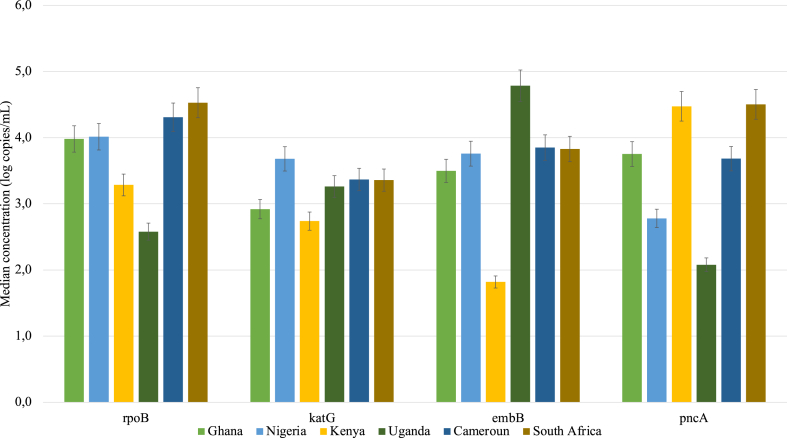
Median concentration (log copies/mL) of genes encoding resistance to first-line TB drugs i.e ethambutol (*embB*), isoniazid (*katG*), rifampicin (*rpoB*), and pyrazinamide (*pncA*) in treated wastewater from the six sub-Saharan African countries.

The lowest median concentrations of ARGs observed in the treated wastewater were observed in Uganda (*rpoB* and *pncA* genes) and Kenya (*embB* and *katG* genes) (Fig. 3). The variation in concentrations of the ARGs associated with resistance to first-line TB drugs in treated wastewater was statistically significant (p ≤ 0.05). Dunn's multiple tests further revealed that this difference was driven by the difference in concentration of *pncA,* and *rpoB* genes between Uganda and South Africa. Furthermore, significant differences were also observed in the concentration of the *katG* gene between Nigeria and Kenya, and lastly concentrations of the *embB* gene between Kenya and Uganda.

#### 3.2.1 Concentration of genes encoding resistance to second-line TB drug in treated wastewater

The genes associated with the resistance to second-line TB drugs were also found in the treated wastewater. The highest median concentrations of these genes in the treated wastewater were from South Africa, for *rrs, alr, fgd1 and atpE* genes encoding resistance to streptomycin, cycloserine, delamanid and bedaquiline respectively, with median concentrations of 4.4(±2,97) log copies/mL, 2.2(±1,49) log copies/mL, 2.7(±2.09) log copies/mL and 3.7(±2,35) log copies/mL respectively. In Kenya, concentrations of the *ddn* gene were the highest ((4.5(±3,32) log copies/mL), in Ghana the *fbiA* gene, (2.5(±2,09) log copies/mL), and the *ethR* gene (3.6(±3,12) log copies/mL) were the highest. Furthermore, concentrations of the *eis* gene were the highest in Nigeria (3.2(±1,18) log copies/mL.

**Fig. 4:**
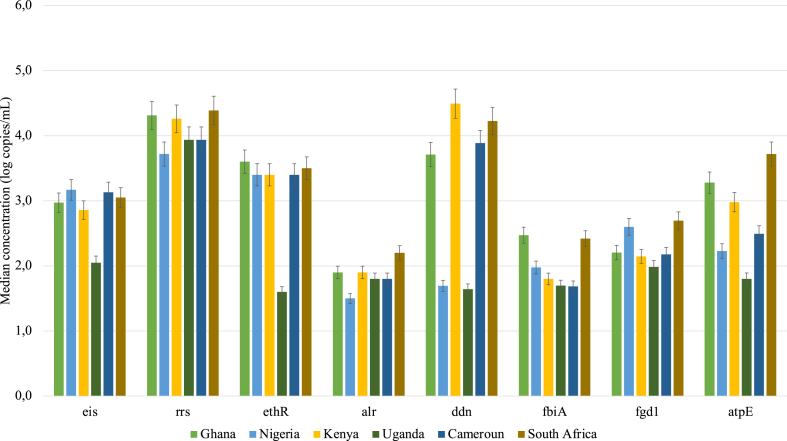
Median concentration (log copies/mL) of selected genes encoding resistance to the second-line TB drugs i.e streptomycin(*rrs*), aminoglycosides (*eis*), ethionamide (*ethR*), cycloserine (*alr*), delaminid (*ddn*, *fgd1* and *fbiA*) and bedaquiline (*atpE*) in treated wastewater from the six sub-Saharan African countries.

In contrast, the lowest concentrations for *eis, ddn, fgd1, atpE* and ethR genes were observed in Uganda (Fig. 4). Furthermore, the lowest concentrations for *alr* and *rrs* genes of 1.5(±1,23) log copies/mL and 3.7(±3,22) log copies/mL respectively were detected in Nigeria. The difference in ARG concentrations across the six countries was observed to be statistically significant (p ≤ 0.05). The within-country comparison also highlighted significant differences between the ARGs, indicating differences in the contribution of WWTPs to the dissemination of the ARGs in each country.

### 3.3 Reduction of TB-related ARGs during wastewater treatment

**Fig. 5:**
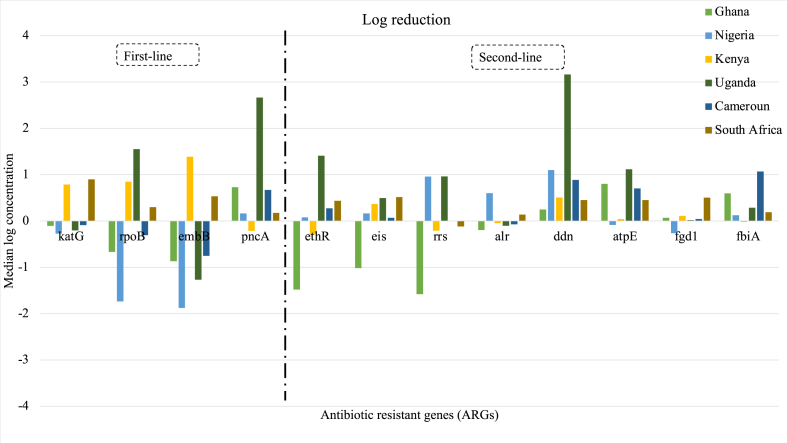
Log reduction of TB-associated ARGs in wastewater treatment plants.

The removal of ARGs varied across different countries. Specifically, when considering the removal of the first line ARGs, the median removal for *katG* was 0.16 log. The highest removal of 0.89 log was achieved at the WWTPs in Durban, South Africa. In contrast, an increase in the concentration of these ARGs was observed in countries such as Ghana, Nigeria, Uganda, and Cameroon (refer to Fig. 5). For example, in Nigeria, there was an increase of 0.27 log in the final effluent compared to the influent.

It is important to note that reduction during wastewater treatment was only observed for *katG* in South Africa and Kenya, with log reductions of 0.89 log and 0.78 log, respectively. In the remaining countries, the concentration of this ARG increased in the treated wastewater compared to the untreated wastewater (Fig. 5). A similar trend was observed for *embB*, with the highest reduction achieved in Kenya. Additionally, the WWTPs in South Africa achieved removal of all ARGs, followed by Kenya, which also achieved removal of all first-line TB ARGs except for *pncA,* which showed an increase in the treated wastewater (−0.21 log). An increase in *pncA* during wastewater treatment was only observed in Kenya (Fig. 5).

The efficiency of removal of ARGs associated with second-line TB drugs also varied among countries. However, treated wastewater samples from Ghana generally had higher concentrations of ARGs compared to untreated wastewater samples, except for *ddn, atpE, fgd1,* and *fbiA* (Fig. 5). The WWTP in Uganda demonstrated the best removal of *ethR, rrs, ddn,* and *atpE*, with log reduction values of 1.4, 0.9, 3.2, and 1.1, respectively. It is worth mentioning that only *ddn* was consistently removed in all countries. The remaining second-line TB drug ARGs showed an increase in concentrations during wastewater treatment in at least one country.

## 4 Discussion

The rise of antibiotic-resistant bacteria is largely due to the widespread use of antibiotics in both humans and animals. These bacteria with resistant traits can spread from human to human, animal to animal, and human to animal and vice versa. They can also be found in environments such as wastewater, which serve as a reservoir for potential pathogens and resistant genes, and contribute to their spread [[19], [20], [21]]. This is supported by the One Health approach to Antimicrobial resistance [22]. The One Health and Global Health concepts incorporate an understanding of the biological elements necessary to comprehend the AMR evolution, such as the microorganisms and vectors involved in its emergence and spread, the host organisms (human or animal), the environments involved, and the socioeconomic and cultural features that facilitate its spread.

The results obtained in this study, which is the detection of ARGs associated with TB treatment in varying concentrations, give a snapshot of the profile of TB resistance within the communities of the selected African countries. The highest concentrations of genes conferring resistance to first-line TB drugs in South Africa, in both untreated and treated wastewater, could be due to the high incidence of TB in the country [5,23]. South Africa has been reported as one of 30 high TB/HIV burden countries [[24], [25]]. The most abundant TB-related ARG detected in South African wastewater samples was the *rpoB* gene (4.8(±2.96) log copies/mL), conferring resistance to rifampicin. Rifampicin is used as the first-line drug for the treatment of drug-susceptible TB in South Africa [26] along with isoniazid (*KatG* gene), ethambutol (*embB* gene) and pyrazinamide (*pncA* gene) [27]. These anti-tubercular compounds, such as rifampicin and pyrazinamide, are not readily degradable and therefore remain persistent in the environment [28], which may explain the high concentration of the genes conferring resistance to these two drugs as compared to isoniazid and ethambutol, which are partially degradable. Magwira et al. (2019) reported that some first-line TB drugs remain unmetabolized and excreted, therefore, end up entering the environment. They conducted a study in Gauteng, South Africa, and found that 20%, 24%, 50%, and 34% of each drug, for every annual usage of isoniazid, rifampicin, ethambutol, and pyrazinamide, respectively, ended up in the environment [28]. Rifampicin is regarded as one of the most effective TB medications and is commonly prescribed to patients [29]. The high concentrations observed for the *rpoB* gene are in agreement with South African clinical data regarding rifampicin resistance. Over 13 000 individuals with rifampicin resistance were reported in 2019 [30], based on only notified and confirmed cases. Nonetheless, it is important to note that the high level of ARG associated with rifampicin resistance may be related to its use in other common infections, such as *Staphylococcus aureus* skin infections, osteoarticular infections and brucellosis [27,[31], [32], [33]]. Other studies in South Africa have reported a high prevalence of pyrazinamide (Mvelase et al., 2022; Ismail et al., 2018) isoniazid and ethambutol (Ismail et al., 2018) resistance. The presence of antibiotic resistance genes (ARGs) for second-line TB drugs, which are also used for other infections, was detected at varying levels in various countries. For instance, the *rrs* gene, conferring resistance to streptomycin and other aminoglycosides such as amikacin and kanamycin (used in TB/multidrug-resistant TB treatment regimens), was found in high concentrations in untreated wastewater from Nigeria and Uganda. These ARGs confer resistance to injectable TB drugs reported in clinical studies [34,35]; Sekyere et al., 2019; Micheni et al., 2022). The high concentrations may also be attributed to the source of the wastewater, i.e. hospital sewage from these countries and also the use of these drugs to treat other common illnesses. Hospitals are widely regarded as a direct source of ARGs [36]; Szekeres et al., 2017). Aminoglycosides are mostly non-metabolized after administration and excreted unchanged via the urine which may also explain the high concentrations of the genes conferring resistance to this group of antibiotics observed. Some studies have reported a range of 75–90% of these drugs being excreted and discharged in their original forms (Tehrani et al., 2021 [37]; Magwira et al., 2019; [16]. Higher concentrations of this gene may also be due to the over usage of these antibiotics for different types of illness, such as *Staphylococcus* infections. *Staphylococcus* infections are very common in South Africa, and they are resistant to common antibiotics [[38], [39], [40], [41]].

Additionally, the highest concentration of the *pncA* gene was detected both in Kenya and Ghana. This aligns with their first-line TB treatment regimen, which includes pyrazinamide as one of the core antibiotics for drug-susceptible TB in these countries. The least abundant ARG was the *alr* gene, conferring resistance to cycloserine. Cycloserine was given group B status by the World Health Organization (WHO) in 2018, the second group in the hierarchy of drugs for resistant TB, and was advised in longer MDR-TB treatment regimens [42]. Extreme-drug resistant TB (XDR-TB) is caused by an *M. tuberculosis* strain with additional resistance to one of the fluoroquinolones or a second-line injectable drug but not both, whereas MDR-TB is caused by *M. tuberculosis* that is resistant to the two most potent fluoroquinolones, isoniazid and rifampicin. The addition of cycloserine is associated with improved MDR-TB treatment success and lower mortality, but it is limited by treatment-associated neuropathy, psychosis, and depression, which forces some patients to discontinue therapy [43,44]. This drug is therefore not commonly used as a core TB drug which may explain the low concentration of this ARG in untreated wastewater.

The results showed that the severity of drug resistance was higher in South Africa than in other parts of the continent. Several factors may contribute to this. For instance, South Africa was the first nation on the African continent to introduce second-line TB treatment in 2001. As a result of their continued use over the years, the country may now be experiencing an increase in drug resistance [4]. The significant difference observed in untreated wastewater concentrations of the *rpoB* gene between Nigeria and South Africa may also be a result of the incident cases reported in these countries. The 2020 WHO TB global report estimated that the incidents of TB in South Africa were 328 000 and 452 000 in Nigeria, a country with a population almost 4 times the population of South Africa [5].

This study highlights key insights based on collected data. For example, the *ddn* gene conferring resistance to the drug delamanid was found to be the most prevalent in untreated wastewater from Cameroon, even though delamanid is not part of the country's second-line treatment regimen [45]. This high concentration suggests the potential spread of delamanid resistance in Cameroon, which may be due to migration, access to the drug through unauthorized channels, or gene transfer in the environment between environmental matrices. In some African countries, antibiotics can be purchased without a prescription, which could also contribute to the detection of these genes [46]. These findings emphasize the need for surveillance tools, such as wastewater-based epidemiology, to support clinical surveillance and provide a clear understanding of the African situation regarding drug-resistant TB infections.

In addition to the untreated wastewater study showing the prevalence of antibiotic resistance to TB in the chosen countries, this study also highlights the potential contribution of wastewater treatment plants to the dissemination of TB-related ARGs based on their presence in the treated final effluent samples. The ARGs conferring resistance to both first-line and second-line TB drugs were abundant in the treated wastewater from all six countries which can be attributed to various factors. Some of these factors include the presence of these genes in viable bacteria capable of surviving the treatment processes in high concentrations, the presence of these genes as nucleic acids in the effluent, and the potential for horizontal gene transfer to result in other bacteria carrying these genes in these conducive environments or potential mobile gene elements (MGEs) carrying these genes [36]. No successful studies have been conducted to isolate these pathogens specifically MTBC from wastewater. Therefore, their survival potential in wastewater needs to be explored. *Mycobacterium* species are commonly found associated with the amoeba in wastewater which may explain their survival in wastewater. Amoeba also acts as a host for carrying these pathogens and resistant genes and MGEs [[47], [48], [49]].

The results of this study also show that the removal of ARGs in WWTPs varies significantly among countries. The variation in ARG removal among countries is likely due to several factors, including the type of WWTP, the treatment process used, and the concentration of ARGs in the influent wastewater. For example, WWTPs that use activated sludge treatment have been shown to be more effective at removing ARGs than WWTPs that use other treatment processes, such as trickling filters or lagoons [50].). Additionally, the concentration of ARGs in the influent wastewater can affect the efficiency of removal. WWTPs that receive wastewater with high concentrations of ARGs may have difficulty removing all the ARGs.

The increase in ARG concentrations in treated wastewater in some countries is a concerning finding. This suggests that WWTPs may be a source of ARGs to the environment. ARGs can be released into the environment from WWTPs through several pathways, including:

ARGs can be released into the environment through the effluent from WWTPs. The effluent from WWTPs is typically treated to remove pathogens and other contaminants, but it is possible that some ARGs may escape removal. ARGs can also be released into the environment through sludge from WWTPs [51,52]. Sludge is the solid material that is removed from wastewater during treatment. Sludge is typically treated to reduce the number of pathogens, but it is possible that some ARGs may survive treatment. ARGs can also be released into the air from WWTPs. This can happen through a number of mechanisms, such as aerosolization. ARGs can be aerosolized during wastewater treatment processes, such as aeration [53]. Aerosolized ARGs can then be released into the air.

The increase in ARG concentrations in treated wastewater, which at times are higher than in untreated wastewater [54,55], may also be due to selective pressures that prevent the growth of antibiotic-susceptible bacteria or promote the selection of mutants. The release of ARGs from WWTPs into the environment is a potential public health risk.

## 5 Study limitations and recommendations

Acquiring representative samples from countries with poor wastewater treatment infrastructures presents challenges and hinders the full potential of current WBE approaches [56]. The possibility of these genes being extracellular or carried by mycobacterial cells or other bacterial species cannot be disregarded. Therefore, further research is necessary to determine whether the genes found in wastewater are extracellular or carried by viable microorganisms and to thoroughly study the viability and infectiousness of these organisms. Long-term studies are also required to evaluate the potential health effects of untreated wastewater and surface water contaminated with wastewater. Adopting WBE as a supplementary surveillance method for drug-resistant TB and populations, especially in low-income countries, is advisable.

Due to various factors such as limited access to healthcare, fear of discrimination and stigmatization due to TB/HIV coinfection, and fear of isolation/hospitalization from DR-TB infection, a significant portion of DR-TB cases are never reported or treated. It is crucial to supplement clinical data with environmental data. Investigating the diversity of carrier bacteria through advanced microbial community analysis to determine infectivity and public health impact is also recommended. Additionally, the presence of ARGs in both treated and untreated wastewater could increase the risk of resistant TB infections for workers at WWTPs. To reduce this risk, it is advised to use personal protective equipment.

## 6 Conclusion

The study provides evidence that genes encoding resistance to anti-TB drugs, both first-line and second-line, are commonly present in untreated wastewater in sub-Saharan Africa, suggesting their potential widespread distribution in the population. South Africa was found to have the highest burden of TB/MDR-TB among the investigated countries, due to the high prevalence of resistance genes related to both first-line and second-line anti-TB drugs. This finding aligns with data from the World Health Organization (WHO). Cameroon was found to have a high concentration of the *ddn* gene, even though the drug associated with it is not included in the country's TB treatment plan. This pattern was also seen in other African nations with resistance genes to delamanid and bedaquiline, two anti-TB drugs not used in their TB treatment. The study also suggests that wastewater treatment facilities in sub-Saharan Africa may contribute to the spread of resistance genes into the environment, as shown by the elevated concentrations of these genes in treated wastewater. These findings emphasize the need for community-level data collection through supplementary surveillance and monitoring, and the importance of wastewater-based epidemiology in disease monitoring alongside clinical information, especially in sub-Saharan Africa where data on drug-resistant TB is limited. Regular and consistent epidemiological surveillance is crucial in reducing the burden of drug-resistant TB and in the development and implementation of relevant policies and interventions.

The findings of this study are also significant for public health because they show that WWTPs may be a source of ARGs to the environment. ARGs can increase the risk of infection with antibiotic-resistant bacteria, which can make it more difficult to treat infections and can lead to longer hospital stays and higher healthcare costs. The findings also highlight the need for better monitoring of ARGs in the environment. This monitoring will help to identify areas where ARGs are present and to track their spread.

## Author contribution statement

Hlengiwe Nombuso Mtetwa: Conceived and designed the experiments; Performed the experiments; Analyzed and interpreted the data; Wrote the paper.

Isaac Dennis Amoah: Conceived and designed the experiments; Analyzed and interpreted the data; Wrote the paper.

Sheena Kumari; Faizal Bux; Poovendhree Reddy: Conceived and designed the experiments; Contributed reagents, materials, analysis tools or data.

## Data availability statement

Data will be made available on request.

## Declaration of competing interest

The authors declare that they have no known competing financial interests or personal relationships that could have appeared to influence the work reported in this paper.
