# Supplementary material for: Surveillance of multidrug-resistant tuberculosis in sub-Saharan Africa through wastewater-based epidemiology
Source: Heliyon. 2023 Jul 21;9(8):e18302. doi: 10.1016/j.heliyon.2023.e18302 (PMC10412881; doi:10.1016/j.heliyon.2023.e18302)
Supplement: Multimedia component 1 [file mmc1.docx]

Supplementary data

Table S1: Target-resistant= genes and their PCR primer sequences

(Mtetwa et al., 2021)

| **Target gene- locus** | **Encoded protein** | **Drug name** | **Primer sequence** | **References** |
| --- | --- | --- | --- | --- |
| *rpoB* | β-Subunit of RNA polymerase | Rifampicin | F:5’-CGAGGTGCCGGTGGAAAC-3’ | Farah Aldour et al. (2018); Pérez-Osorio et al. (2012); Rodwell et al. (2014); Nguyen, 2016 |
|  |  |  | R:5’-GTCGTCGTGCTCCAGGAAGG-3’ |  |
| *KatG* | catalase-peroxidase | Isoniazid | F:5’-GAGCCCGATGAGGTCTATTG-3’ | Farah Aldour et al. (2018); Pérez-Osorio et al. (2012); Rodwell et al. (2014); Nguyen, 2016 |
|  |  |  | R:5’-GTCCTTGGCGGTGTATTGC-3’ |  |
| *inhA* | Enoyl ACP reductase |  | F:5’-GAGCGTAACCCCAGTGCGAA-3’ | Rodwell et al. (2014); Nguyen, 2016 |
|  |  |  | R:5’-TCCGGTAACCAGGACTGAAC-3’ |  |
| *embB* | arabinosyltransferase | Ethambutol | F:5’-CATGTCATCGGCGCGAATTCG-3’ | Nguyen, 2016 |
|  |  |  | R:5’-TGGCAGGCGCATCCACAGACT-3’ |  |
| *PncA* | pyrazinamidase | Pyrazinamide | F:5’-GACGTATGCGGGCGTTGA-3’ | Farah Aldour et al. (2018); Pérez-Osorio et al. (2012) |
|  |  |  | R:5’-CCATCAGGAGCTGCAAACCA-3’ |  |
| *gyrA* | DNA gyrase subunit A | Ofloxacin, Moxifloxacin | F:5’-GGTGCTCTATGAAATGTTCG-3’ | Rodwell et al. (2014) |
|  |  |  | R:5’-GCTTCGGTGTACCTCATCG-3’ |  |
| *gyrB* | DNA gyrase subunit B |  | F:5’-CGATGTTCCAGGCGATACTT-3’ | Rodwell et al. (2014) |
|  |  |  | R:5’-ATCTTGTGGTAGCGCAGCTT-3’ |  |
| *rrs* | 16S ribosomal RNA | Kanamycin, Amikacin | F:5’-GTAATCGCAGATCAGCAACG-3’ | Rodwell et al. (2014); Nguyen, 2016 |
|  |  |  | R:5’-TTTTCGTGGTGCTCCTTAGAA-3’ |  |
| *eis* | Aminoglycoside acetyltransferase | Amikacin, Kanamycin | F:5’-AAATTCGTCGCTGATTCTCG-3’ | Rodwell et al. (2014) |
|  |  |  | R:5’-CGCGACGAAACTGAGACC-3’ |  |
| *rpsL* or *rrs* | 30S ribosomal protein S12/16S ribosomal RNA | Streptomycin | F:5’-GCGCCCAAGATAGAAAG-3’ | Nguyen, 2016 |
|  |  |  | R:5’-CAACTGCGATCCGTAGA-3’ |  |
| *ddn* | deazaflavin-dependent nitroreductase | Delamanid | F:5’-CGAGCGCACCGACCAGAGC-3’ | Yang et al. (2018) |
|  |  |  | R:5’-GCATGGCCCGCAGGTGGACAA-3’ |  |
| *fbiA* | 2-phospho-L-lactate transferase |  | F:5’-GCGGTTCTGTTGTGGTTGGG-3’ | Yang et al. (2018) |
|  |  |  | R:5’-CCGATGACGGGCAGGATCTCGATGG-3’ |  |
| *fgd1* | F420-dependent glucose-6-phosphate |  | F:5’-CGTGGCCGCGAGCGAGGTGAA-3’ |  |
|  |  |  | R:5’-CGCCCGAACCGTCAACAACACTGG-3’ |  |
| *Rv0678* | hypothetical protein | Bedaquiline | F:5’-GTATCCAGGCACGCTTGA-3’ | Yang et al. (2018) |
|  |  |  | R:5’-CCCCACAATCGATAACC-3’ |  |
| *atpE* | ATP synthase subunit C |  | F:5’-GTACTTCAGCCAAGCGATGG-3’ | Yang et al. (2018) |
|  |  |  | R:5’-CCGTTGGGAATGAGGAAGTTG-3’ |  |
| *ethA* | monooxygenase EthA | Ethionamide | F:5′-CCTGGCAGCTTACTACGTGTC-3 | Tan et al. (2017) |
|  |  |  | R:5′-CGGCATCATCGTCGTCTG-3′ |  |
| *ethR* | HTH-type transcriptional repressor |  | F:5′-TTTTCCAGGATGGCGTAGC-3′ | Tan et al. (2017) |
|  |  |  | R:5′-CCGACCGGATCGTCAACA-3′ |  |
| *alr* | alanine racemase | Cycloserine | F:5’-GAAAATAAAAGACACGCCTACTTTCGCTCCA-3’ | Chen et al. (2017) |
|  |  |  | R:5’-GACATCCATCGCCATGGCAATACCCTT-3’ |  |

Table S2: Median (SD) log copies/mL achieved for the various antimicrobial-resistance genes in the three wastewater treatment plants (Ghana, Nigeria & Kenya)

|  | Ghana | | | | Nigeria | | | | Kenya | | | | |
| --- | --- | --- | --- | --- | --- | --- | --- | --- | --- | --- | --- | --- | --- |
|  | Influent | | Effluent | | Influent | | Effluent | | Influent | | Effluent | | |
|  | Median (±SD) | Range | Median (±SD) | Range | Median (±SD) | Range | Median (±SD) | Range | Median (±SD) | Range | Median (±SD) | Range |  |
| *katG* | 2.8(±2,33) | 0.47-2.13 | 2.9(±1,53) | 1.37-4.27 | 3.4(±3,50) |  | 3.7(±2,47) | 1.23-6.17 | 3.5(±2,69) | 0.81-6.19 | 2.7(±1,83) | 0.87-4.53 |  |
| *rpoB* | 3.3(±1.73) | 1.57-5.03 | 4.0(±2.15) | 1.85-6.15 | 2.3(±0.93) | 1.37-3.23 | 4.0(±2.49) | 1.51-6.49 | 4.1(±2.30) | 1.8-6.40 | 3.3(±2.88) | 0.42-6.18 |  |
| *embB* | 2.6(±1,88) | 0.72-4.48 | 3.5(±2,22) | 1.28-5.72 | 1.9(±1,45) | 0.45-3.35 | 3.8(±2,64) | 1.16-6.44 | 3.2(±1,30) | 1.90-4.5 | 1.8(±2,97) | -1.17-4.77 |  |
| *pncA* | 4.5(±3,42) | 1.08-7.92 | 3.8(±2,22) | 1.58-6.02 | 2.9(±2,02) | 0.88-4.92 | 2.8(±2,64) | 0.16-5.44 | 4.3(±2,90) | 1.4-7.2 | 4.5(±2,97) | 2.23-6.77 |  |
| *eis* | 2.0(±0,45) | 1.55-2.45 | 3.0(±2,69) | 0.31-5.69 | 3.3(±0,45) | 2.85-3.75 | 3.2(±1,18) | 2.02-4.38 | 3.2(±2,62) | 0.58-5.82 | 2.9(±3,32) | -0.42-6.22 |  |
| *rrs* | 2.7(±2,25) | 0.45-4.95 | 4.3(±3,45) | 0.85-7.75 | 4.7(±3,51) | 1.19-8.21 | 3.7(±3,22) | 0.48-6.92 | 4.1(±4,16) | -0.06-8.26 | 4.3(±3,66) | 0.64-7.96 |  |
| *alr* | 1.8(±0,83) | 0.94-2.63 | 1.9(±0,72) | 1.18-2.62 | 2.1(±1,93) | 0.17-4.03 | 1.5(±1,23) | 0.27-2.73 | 1.8(±1,14) | 0.66-2.94 | 1.9(±1,01) | 0.89-2.91 |  |
| *ethR* | 2.1(±1,05) | 1.05-3.15 | 3.6(±3,12) | 0.48-6.72 | 3.5(±2,66) | 0.84-6.16 | 3.4(±2,83) | 0.57-6.23 | 3.1(±2,02) | 1.08-5.12 | 3.4(±1,77) | 1.63-5.17 |  |
| *ddn* | 4.0(±3,44) | 0.56-7.44 | 3.7(±2,69) | 1.01-6.39 | 2.8(±2,34) | 0.46-5.14 | 1.7(±1,18) | 0.52-2.88 | 5.0(±4,22) | 0.78-9.22 | 4.5(±3,32) | 1.18-7.82 |  |
| *Fgd1* | 2.3(±0,15) | 2.15-2.45 | 2.2(±1,23) | 0.97-3.43 | 2.3(±0.45) | 1.85-2.75 | 2.6(±1.41) | 1.19-4.01 | 2.3(±1.23) | 1.07-3.53 | 2.1(±0.75) | 1.35-2.85 |  |
| *fbiA* | 3.1(±1,53) | 1.57-4.63 | 2.5(2,09) | 0.41-4.59 | 2.1(±1.57) | 0.53-3.67 | 2.0(±1.13) | 0.87-3.13 | 1.8(±1.05) | 0.75-2.85 | 1.8(±0.15) | 1.95 |  |
| *atpE* | 3.7(±2,21) | 1.49-5.91 | 3.3(±1,80) | 1.5-5.10 | 2.1(±1,53) | 0.57-3.63 | 2.2(±0,86) | 1.34-3.06 | 3.0(±2,99) | 0.01-5.99 | 3.0(±3,32) | -0.32-6.32 |  |
|  |  |  |  |  |  |  |  |  |  |  |  |  |  |

Table S3: Median (SD) log copies/mL achieved for the various antimicrobial-resistance genes in the three wastewater treatment plants (Uganda, Cameroon and South Africa

|  | Uganda | | | | Cameroon | | | | South Africa | | | |
| --- | --- | --- | --- | --- | --- | --- | --- | --- | --- | --- | --- | --- |
|  | Influent | | Effluent | | Influent | | Effluent | | Influent | | Effluent | |
|  | Median (±SD) | Range | Median (±SD) | Range | Median (±SD) | Range | Median (±SD) | Range | Median (±SD) | Range | Median (±SD) | Range |
| *katG* | 3.1(±0,75) | 2.35-3.85 | 3.3(±2,50) | 0.80-5.80 | 3.3(±2,30) | 1.00-5.60 | 3.4(±2,09) | 1.31-5.49 | 4.3(±2,77) | 1.53-7.07 | 3.4(±2,06) | 1.34-5.46 |
| *rpoB* | 4.1(±2.85) | 1.25-6.95 | 2.6(±2.16) | 0.44-4.76 | 4.0(±3.32) | 0.68-7.32 | 4.3(±1.45) | 2.85-5.75 | 4.8(±2.96) | 1.84-7.76 | 4.59(±3.86) | 0.73-8.45 |
| *embB* | 3.5(±2,88) | 0.62-6.38 | 4.8(±1,71) | 3.09-6.51 | 3.1(±1,60) | 1.50-4.70 | 3.9(±2,60) | 1.30-6.50 | 4.4(±3,10) | 1.30-7.50 | 3.8(±4,10) | 0.30-7.90 |
| *pncA* | 4.7(±3,11) | 1.59-7.81 | 2.1(±1,71) | 0.39-3.81 | 4.4(±3,11) | 1.29-7.51 | 3.7(±3,56) | 0.14-7.26 | 4.7(±3,39) | 1.31-8.09 | 4.5(±4,10) | 0.40-8.60 |
| *eis* | 2.5(±1,05) | 1.45-3.55 | 2.0(±1,20) | 0.80-3.20 | 3.2(±2,30) | 0.90-5.50 | 3.1(±2,43) | 0.67-5.53 | 3.6(±3,28) | 0.32-6.88 | 3.0(±2,79) | 0.21-5.79 |
| *rrs* | 4.9(±3,81) | 1.09-8.71 | 3.9(±2,56) | 1.34-6.46 | 3.9(±4,09) | -0.19-7.99 | 3.9(±2,57) | 1.33-6.47 | 4.3(±3,51) | 0.79-7.81 | 4.4(±2,97) | 1.43-7.37 |
| *alr* | 1.7(±1,43) | 0.27-3.13 | 1.8(±0,10) | 1.70-1.90 | 1.7(±1,42) | 0.28-3.12 | 1.8(±1,15) | 0.65-2.95 | 2.4(±2,02) | 0.38-4.42 | 2.2(±1,49) | 0.71-3.69 |
| *ethR* | 3.0(±2,63) | 0.37-5.63 | 1.6(±0,75) | 0.85-2.35 | 3.7(±2,60) | 1.1-6.3 | 3.4(±3,42) | -0.02-6.82 | 3.9(±2,93) | 0.97-6.83 | 3.5(±2,93) | 0.57-6.43 |
| *ddn* | 4.8(±3,25) | 1.55-8.05 | 1.6(±1,20) | 0.40-2.80 | 4.8(±3,67) | 1.13-8.47 | 3.9(±2,43) | 1.47-6.33 | 4.7(±1,64) | 3.10-6.30 | 4.2(±2,79) | 1.41-6.99 |
| *Fgd1* | 2.0(±1.05) | 0.95-3.05 | 2.0(±1.23) | 0.77-3.23 | 2.2(±0.45) | 1.75-2.65 | 2.2(±1.15) | 1.05-3.35 | 3.2(±1.85) | 1.35-5.05 | 2.7(±2.09) | 0.61-4.79 |
| *fbiA* | 2.0(±1.90) | 0.10-3.9 | 1.7(±1.42) | 0.28-3.12 | 2.8(±2.28) | 0.52-5.08 | 1.7(±1.30) | 0.40-3.00 | 2.6(±1.81) | 0.79-4.41 | 2.4(±1.15) | 1.25-3.55 |
| *atpE* | 2.9(±-0,07) | 2.83-2.97 | 1.8(±1,32) | 0.48-3.12 | 3.2(±2,31) | 0.89-5.51 | 2.5(±0,91) | 1.59-3.41 | 4.5(±3,74) | 0.76-8.24 | 3.7(±2,35) | 1.35-6.05 |
|  |  |  |  |  |  |  |  |  |  |  |  |  |

References

1. Farah Aldour, M.S.M., Elhussein, A.R.M., Elkhidir, I.M., Tayeib, S.E., Mohammed Khair, O., Mohamed, N.S. and Enan, K.A., 2018. Detection of Drug Resistant Genes of Mycobacterium tuberculosis in Sudanese Tuberculosis Patients in Khartoum State Using Multiplex PCR. <http://repo.nusu.edu.sd/xmlui/handle/123456789/132>
2. Yang, J.S., Kim, K.J., Choi, H. and Lee, S.H., 2018. Delamanid, bedaquiline, and linezolid minimum inhibitory concentration distributions and resistance-related gene mutations in multidrug-resistant and extensively drug-resistant tuberculosis in Korea. *Annals of laboratory medicine*, *38*(6), p.563. <https://doi.org/10.3343/alm.2018.38.6.563>
3. Tan, Y., Su, B., Zheng, H., Song, Y., Wang, Y. and Pang, Y., 2017. Molecular characterization of prothionamide-resistant Mycobacterium tuberculosis isolates in southern China. *Frontiers in Microbiology*, *8*, p.2358. <https://doi.org/10.3389/fmicb.2017.02358>
4. Rodwell, T.C., Valafar, F., Douglas, J., Qian, L., Garfein, R.S., Chawla, A., Torres, J., Zadorozhny, V., Kim, M.S., Hoshide, M. and Catanzaro, D., 2014. Predicting extensively drug-resistant Mycobacterium tuberculosis phenotypes with genetic mutations. *Journal of clinical microbiology*, *52*(3), pp.781-789. <https://doi.org/10.1128/JCM.02701-13>
5. Nguyen, L., 2016. Antibiotic resistance mechanisms in M. tuberculosis: an update. *Archives of toxicology*, *90*(7), pp.1585-1604. <https://doi.org/10.1007/s00204-016-1727-6>
6. Pérez-Osorio, A.C., Boyle, D.S., Ingham, Z.K., Ostash, A., Gautom, R.K., Colombel, C., Houze, Y. and Leader, B.T., 2012. Rapid identification of mycobacteria and drug-resistant Mycobacterium tuberculosis by use of a single multiplex PCR and DNA sequencing. *Journal of clinical microbiology*, *50*(2), pp.326-336. <https://doi.org/10.1128/JCM.05570-11>
